# Comparison of the intestinal flora of wild and artificial breeding green turtles (*Chelonia mydas*)

**DOI:** 10.3389/fmicb.2024.1412015

**Published:** 2024-05-30

**Authors:** Xin Niu, Liu Lin, Ting Zhang, Xiaoyu An, Yupei Li, Yangfei Yu, Meiling Hong, Haitao Shi, Li Ding

**Affiliations:** ^1^Ministry of Education Key Laboratory for Ecology of Tropical Islands, Key Laboratory of Tropical Animal and Plant Ecology of Hainan Province, College of Life Sciences, Hainan Normal University, Haikou, China; ^2^Hainan Sansha Provincial Observation and Research Station of Sea Turtle Ecology, Sansha, China; ^3^Marine Protected Area Administration of Sansha City, Sansha, China

**Keywords:** *Chelonia mydas*, artificial breeding, green turtles, pathogen detection, microbial composition, reintroduction

## Abstract

Gut microbes are pivotal reference indicators for assessing the health status of animals. Before introducing artificially bred species into the wild, examining their gut microbe composition is crucial to help mitigate potential threats posed to wild populations. However, gut microbiological trait similarities between wild and artificially bred green turtles remain unexplored. Therefore, this study compared the gut microbiological characteristics of wild and artificially bred green turtles (*Chelonia mydas*) through high-throughput Illumina sequencing technology. The α-diversity of intestinal bacteria in wild green turtles, as determined by Shannon and Chao indices, significantly surpasses that of artificial breeding green turtles (*p* < 0.01). However, no significant differences were detected in the fungal α-diversity between wild and artificially bred green turtles. Meanwhile, the β-diversity analysis revealed significant differences between wild and artificially bred green turtles in bacterial and fungal compositions. The community of gut bacteria in artificially bred green turtles had a significantly higher abundance of Fusobacteriota including those belonging to the *Paracoccus*, *Cetobacterium*, and *Fusobacterium* genera than that of the wild green turtle. In contrast, the abundance of bacteria belonging to the phylum Actinobacteriota and genus *Nautella* significantly decreased. Regarding the fungal community, artificially bred green turtles had a significantly higher abundance of *Fusarium*, *Sterigmatomyces*, and *Acremonium* and a lower abundance of *Candida* and *Rhodotorula* than the wild green turtle. The PICRUSt2 analyses demonstrated significant differences in the functions of the gut bacterial flora between groups, particularly in carbohydrate and energy metabolism. Fungal functional guild analysis further revealed that the functions of the intestinal fungal flora of wild and artificially bred green turtles differed significantly in terms of animal pathogens-endophytes-lichen parasites-plant pathogens-soil saprotrophs-wood saprotrophs. BugBase analysis revealed significant potential pathogenicity and stress tolerance variations between wild and artificially bred green turtles. Collectively, this study elucidates the distinctive characteristics of gut microbiota in wild and artificially bred green turtles while evaluating their health status. These findings offer valuable scientific insights for releasing artificially bred green turtles and other artificially bred wildlife into natural habitats.

## 1 Introduction

Sea turtles, known as “living fossils,” play a pivotal role in sustaining marine ecosystems and are widely recognized as a “flagship species” and “umbrella species” for marine organism conservation (Bouchard and Bjorndal, 2000; Senko et al., 2022). Green turtles (*Chelonia mydas*) serve as indicators of marine ecosystem health and are critical in preventing overgrowth of seagrass bed (Chaloupka et al., 2008; Page-Karjian et al., 2020). However, overfishing of eggs and adult specimens has led to a significant decline in green sea turtle population (Bjorndal et al., 2000; Meylan et al., 2022) with the adult population in the South China Sea standing at <2,000 (Wu, 2023). Accordingly, the Action Plan for the Conservation of Sea Turtles in China (2019–2033) advocates for investigating new strategies to replenish wild turtle populations through artificial breeding. Indeed, artificial breeding is crucial in conserving endangered sea turtle populations (Hong et al., 2022), having demonstrated efficacy in expanding, rewilding, releasing, and restoring wild green turtle populations.

Successful artificial breeding programs have been implemented for green turtles globally, including United States (Florida), Japan, and the Cayman Islands (British), serving purposes from population restoration to commercial exploitation (Barbanti et al., 2019). For instance, the Cayman Turtle Farm has contributed significantly to restoring natural populations by releasing green turtles (Barbanti et al., 2019). Similarly, the Guangdong Huidong Sea Turtle National Nature Reserve in China has extensively researched artificial breeding techniques for green turtles (Chen et al., 2007; Ye et al., 2020), resulting in the first reported successful breeding of green turtle offspring in full captivity (Xia et al., 2017). Although artificial breeding significantly contributes to wild population recovery (Hong et al., 2022), caution is essential when planning releases to safeguard the health, behavioral competence, and genetics of wild populations (Kock et al., 2010). China encounters challenges in artificially releasing and recovering endangered species wild populations (Xia and Gu, 2012). For example, attempts have been made to release yellow-throated turtles (*Mauremys mutica*) and Chinese pomfret (*Pampus sinensis*) via artificial breeding. However, enhancing pre-release assessments and post-release monitoring is imperative, while the effect of these releases on wild population recovery remains unclear (Hong et al., 2022).

Breakthroughs in biotechnology may lead to gut microbiome sampling and analysis to become standard protocols for monitoring the health of artificially bred animals (Ahasan et al., 2017). This parallels the widespread adoption of blood chemistry analysis (Franco-Duarte et al., 2019). Lewbart et al. (2014) utilized blood biochemical indicators to evaluate the health status of green turtles for early disease diagnosis and prevention during breeding. However, pathogenic bacterial infections pose a significant threat to the health of green turtles (Ye et al., 2022). Therefore, based solely on blood biochemical indicators, assessing the health status of sea turtles before their release is challenging. Hence, exploring more comprehensive assessment methods to enhance the conservation and recovery of endangered sea turtle populations is crucial.

The gut microbial composition of reptiles varies based on their health status, offering potential reference indicators for assessing animal health (Ma et al., 2008; Ahasan et al., 2017; Zhong et al., 2022; Forbes et al., 2023). Studies on the intestinal microbes in green turtles have recently garnered attention. Scheelings et al. (2020) explored the relationship between green sea turtle gut bacteria and evolution, while Ahasan et al. (2017) found higher bacterial diversity and abundance in wild-caught green turtles than that in stranded sea turtles. Furthermore, food reportedly affects the dominant phyla of gut microorganisms in green turtles (Bloodgood et al., 2020). Insights into the core microbiome of gastrointestinal tract of the sea turtle can enhance the understanding of their health and aid in managing rehabilitation efforts (Flint et al., 2010; Ahasan et al., 2017; McNally et al., 2021).

The association between changes in the gut microbiome and disease susceptibility is well-established across vertebrates and invertebrates (Costa et al., 2012). Opportunistic pathogens might inhabit the gut microbiota and colonize the gastrointestinal tract of immunocompromised animals (Owens et al., 2008; Li et al., 2015). Therefore, analyzing the gut microbiome of an animal can provide insights into its health status (Apprill et al., 2017; Bloodgood et al., 2020). Additionally, detecting gut microbial species is crucial when releasing breeding populations. A study provided scientific guidance for releasing artificially bred *Alligator sinensis* into the wild by detecting their gut microbial species, thus mitigating threats to wild populations (Ma et al., 2008). Similarly, another study aimed to minimize influence on wild bird populations by assessing pathogens before releasing confiscated birds (Cruz et al., 2021). However, release of many turtles lack an effective risk assessment of the pathogen-carrying status of the animals being released (Wu, 2023). This poses a threat to the health of sea turtle populations, particularly green turtles, which have extensive migration routes. Releasing many green turtles carrying pathogens may significantly affect the safety of turtle populations and endanger marine ecosystems. Therefore, ensuring the safety of sea turtle populations and marine ecological security necessitates avoiding the release of large number of green turtles carrying pathogens.

In this study, high-throughput sequencing was used to compare the gut microbiological traits of wild and artificially bred green turtles. The primary objective of this study is to assess the health status of artificially bred green turtles and offer scientific guidance for their release into the wild.

## 2 Materials and methods

### 2.1 Sample collection

This study involved 20 green turtles, including 6 wild *C. mydas* from the Hainan Sansha Provincial Observation and Research Station of Sea Turtle Ecology in Sansha, China, and 14 artificially bred *C. mydas* (AC) from the Polar Marine Park, China. We recorded basic information, including body weight, dorsal carapace length, and width (Supplementary Table S1). Cloacal sampling, widely used in studies on gut microbes in amphibians (Barbosa et al., 2016; Ganz et al., 2017; Merkeviciene et al., 2017), was selected for its simplicity and efficiency in simultaneously obtaining samples from all individuals (Stanley et al., 2015). For green turtles, this method involved inserting the entire swab head into the cloaca, applying gentle pressure in a circular motion and rubbing the inner circumference two to three times. The swab head was broken off, placed into a sterile 5 mL freezing tube, and preserved at −80°C. Animal welfare and experiments were approved by the Animal Research Ethics Committee of Hainan Provincial Education Center for Ecology and Environment, Hainan Normal University (HNECEE-2023-006).

### 2.2 DNA extraction and PCR amplification

Microbial genomic DNA was extracted from cloacal samples using a MagAttract Power Soil Pro DNA Kit (QIAGEN, Hilden, Germany), according to the manufacturer’s instructions. The quality and concentration of DNA were determined using 1.0% agarose gel electrophoresis and a NanoDrop2000 spectrophotometer (Thermo Scientific, United States); DNA was stored at −80°C until subsequent utilization. The V3–V4 highly variable portion of the bacterial 16S rRNA gene and the ITS1 region of the fungal ITS gene were amplified using an ABI GeneAmp® 9,700 PCR Thermal Cycler (GeneAmp 9,700, ABI, United States). For bacterial amplification, primers 338F (5’-ACTCCTACGGGGAGGCAGCAG-3′) and 806R (5’-GGACTACHVGGGTWTCTAAT-3′) were employed (Liu et al., 2016). The primers for the ITS1 region of the fungal ITS gene were 5’-CTTGGTCATTTAGAGAGGAAGTAA-3′ and 5’-GCTGCGTTCTTCATCGATGC-3′ (Qu et al., 2022). PCRs were conducted three times per sample in 20 μL reactions. The PCR mixture comprised Taq Pro Multiplex DNA Polymerase (10 μL), template DNA (10 ng), and each primer (5 μM, 0.8 μL), which was adjusted to 20 μL of ddH_2_O. PCR amplification cycling conditions comprised an initial denaturation at 95°C for 3 min, followed by 29 cycles for bacteria and 35 for fungi. The denaturation was conducted at 95°C for 30 s, followed by annealing at 53°C for bacteria and 55°C for fungi, extension at 72°C for 30 s, and a final single extension at 72°C for 10 min, concluding at 4°C. Subsequently, the PCR product was extracted from a 2% agarose gel, purified using the PCR Clean-Up Kit (YuHua, Shanghai, China) following the manufacturer’s instructions, and quantified using a Qubit 4.0 (Thermo Fisher Scientific, United States).

### 2.3 Illumina MiSeq sequencing

The purified amplicons were pooled in equimolar quantities and sequenced using a paired-end approach on an Illumina PE300 platform (Illumina, San Diego, CA, United States), following the standard protocols of Majorbio Bio-Pharm Technology Co. Ltd. (Shanghai, China). The raw sequencing reads were deposited in the NCBI Sequence Read Archive database (accession Number: SRP497708, SRP497709).

### 2.4 Processing of sequencing data

The raw sequences underwent fastp software quality control (Chen et al., 2018) and were spliced using FLASH software (Magoč and Salzberg, 2011). To enhance read quality, we filtered bases with a quality value of ≤20 from the ends of the reads. We implemented a 50 bp window and trimmed bases from the back end if the average quality value within the window was <20. Additionally, we excluded reads shorter than 50 bp and those containing N-bases. Paired-end reads were merged into a single sequence based on the overlapping relationship between the reads, with a minimum overlap length of 10 bp. The maximum mismatch ratio of 0.2 was allowed in the overlapping region of merged sequences, and non-conforming sequences were filtered. Samples were distinguished based on the barcode and the primer sequences at the first and last end, adjusting the sequence orientation. The barcode allowed zero mismatches, while up to two primer mismatches were permitted. Operational taxonomic units (OTUs) were clustered at a 97% similarity threshold, and chimeras were removed using UPARSE v7.1 software (Stackebrandt and Goebel, 1994; Edgar, 2013). More information on UPARSE can be found at http://drive5.com/uparse/. Sequences annotated to the chloroplast and mitochondrial sequences were removed from all samples. Taxonomic annotation of OTU was conducted using the Silva 16S rRNA (v138) and ITS (Unite v.8.0) gene databases. The RDP classifier (Wang et al., 2007) (http://rdp.cme.msu.edu/, version 2.11) was employed with a confidence threshold of 70%. The community composition of each sample was then determined at various taxonomic levels.

### 2.5 Ecological and statistical analyses

We utilized Mothur (v.1.30.2, University of Michigan, Ann Arbor, MI, United States) to generate sparse curves, assessing the sequencing depth adequacy to cover the estimated number of OTUs at 97% sequence similarity (Schloss et al., 2009). Subsequently, the data were analyzed using Mothur for α-diversity indices, evaluating bacterial and fungal community abundance and diversity. *t*-tests were used to assess α-diversity indices under conditions of normal distribution and homogeneity of data. Otherwise, Wilcoxon rank-sum tests were used to assess differences in α-diversity. β-diversity was assessed to determine similarity in microbial community structure across samples using principal coordinate analysis (PCoA), employing the Bray–Curtis distance algorithm (Schloss et al., 2009). OTU Venn diagrams were generated using the BASE and VEGAN packages in R (Ji et al., 2017). Analysis of community structure at the phylum and genus levels was conducted using the PANDAS package in Python (v.2.7). We utilized the data table from the tax_summary_a folder for analysis. A bar chart was generated, merging relative abundances <1% into the “others” category (Ji et al., 2017). Significant differences between wild and artificially bred green turtles were calculated based on data distribution using the Wilcoxon rank-sum test (*p* < 0.05). Linear discriminant analysis (LDA) was conducted on samples grouped under different conditions, with a significance level set at a Wilcoxon *p*-value of <0.05 and an LDA score of >3.5 (Guerrero-Preston et al., 2016).

We employed PICRUSt2 to predict functional differences in the gut bacterial communities of wild and artificially bred green turtles, leveraging 16S rRNA gene sequences to predict KEGG immediate homolog (KO) functional profiles (Tang et al., 2018). The fungal functional guild (FUNGuild) was also used to predict the functional differences in the intestinal fungal ecology of wild and artificially bred green turtles (Schmidt et al., 2019). Bacterial communities were analyzed using BugBase (Lucas et al., 2018)—a microbiome tool that identifies phenotypic levels and predicts microbial traits. BugBase normalizes OTUs based on the number of predicted 16S copies and uses a precalculated file for predictions. Relative abundance shifts between wild and artificially bred green turtles were compared using the Wilcoxon rank-sum test, with significance at *p* < 0.05.

## 3 Results

### 3.1 Comparison of the intestinal bacteria of wild and artificial breeding green turtles

#### 3.1.1 rRNA sequencing analysis

The bacterial assay yielded 53,255–75,193 quality-filtered sequences obtained per sample, totaling 1,329,090 sequences (408,355 reads in wild green turtles and 920,735 in artificially bred green turtles). Sequences had a length distribution of 409–428 bp (mean: 417 bp) (Supplementary Table S2). All samples were sparse to 45,256 for bacterial sequencing (Supplementary Figure S1). Sparse curves reached the saturation stage, indicating that adequate sampling depth was achieved for each sample.

#### 3.1.2 α- and β-diversity analyses

**Figure 1:**
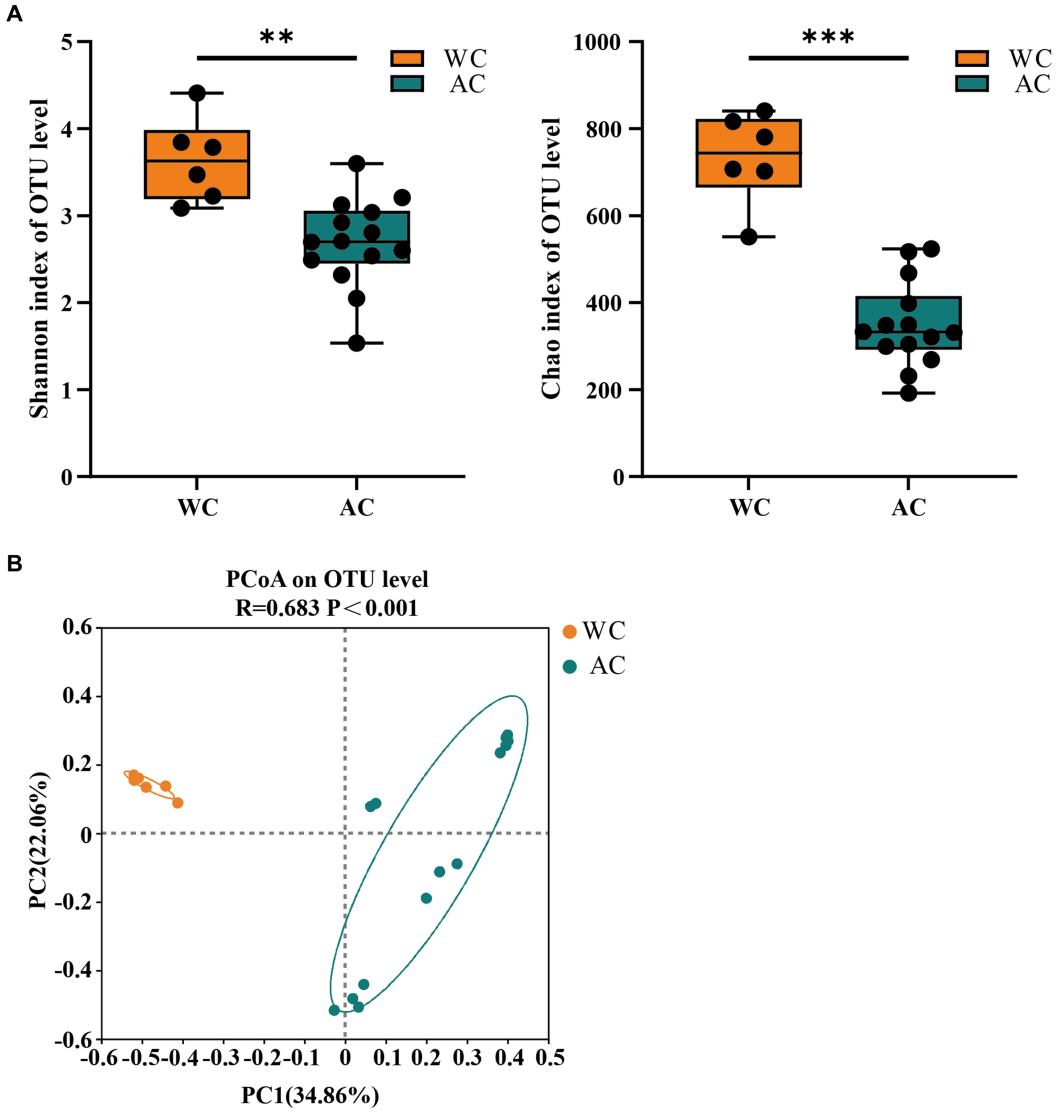
The α-diversity and β-diversity of gut bacteria of the wild (WC) and artificial breeding (AC) green turtles. **(A)** Shannon and Chao data were not significant (*p* > 0.05) as determined via S-W and A-D tests, with normally distributed data. α-diversity of bacteria was measured via Shannon and Chao indices. *p*-values indicate the confidence level of statistical analyses, with *p* < 0.05 indicating statistically significant differences. ** represents *p* < 0.01 and *** represents *p* < 0.001. **(B)** Principal component analysis (PCoA) plot of Beta diversity based on bacterial communities in anal swab samples of green turtles (*Chelonia mydas*). The principal coordinates (PC1 and PC2) are depicted in the axes, with percentages in parentheses indicating their relative contributions. Analysis was conducted using ANOSIM ANOVA. *R*-values range (−1 to 1), where *R*-values close to 0 indicate no significant differences between and within groups, while values close to 1 indicate greater between-group differences than within-group differences. *p*-values indicate the confidence level for statistical analyses, with *p* < 0.05 indicating a statistically significant difference.

The bacterial community evenness (Shannon index) and richness (Chao index) of wild green turtles were significantly higher than that of artificially bred green turtles (*p* < 0.01; Figure 1A). ANOSIM analysis revealed significant differences between the gut bacterial communities of wild and artificially bred green turtles (*R* = 0.683, *p* < 0.001; Figure 1B).

#### 3.1.3 Microbial community composition and predicted functional analysis

Venn diagrams illustrated the shared and distinct bacterial microbiota of wild and artificially bred green turtles (Supplementary Figure S2). In the bacterial community, 2,408 OTUs were found, with 483 (20.06%) shared. Among these shared OTUs, 216 belonged to Proteobacteria (44.72%), 107 belonged to Bacteroidota (22.15%), and 91 belonged to Firmicutes (18.84%). Additionally, 1,164 OTUs (48.34%) were unique to wild green turtles. Among these, 387 belonged to Proteobacteria (33.25% unique), 267 belonged to Bacteroidota (22.94% unique), and 126 belonged to Firmicutes (10.82% unique). In the artificially bred turtles, 761 OTUs were identified as unique, comprising 31.60% of the total. Among these, 282 belonged to the phylum Proteobacteria (30.06% unique), 181 belonged to Bacteroidota (23.78% unique), and 149 belonged to Firmicutes (19.58% unique).

**Figure 2:**
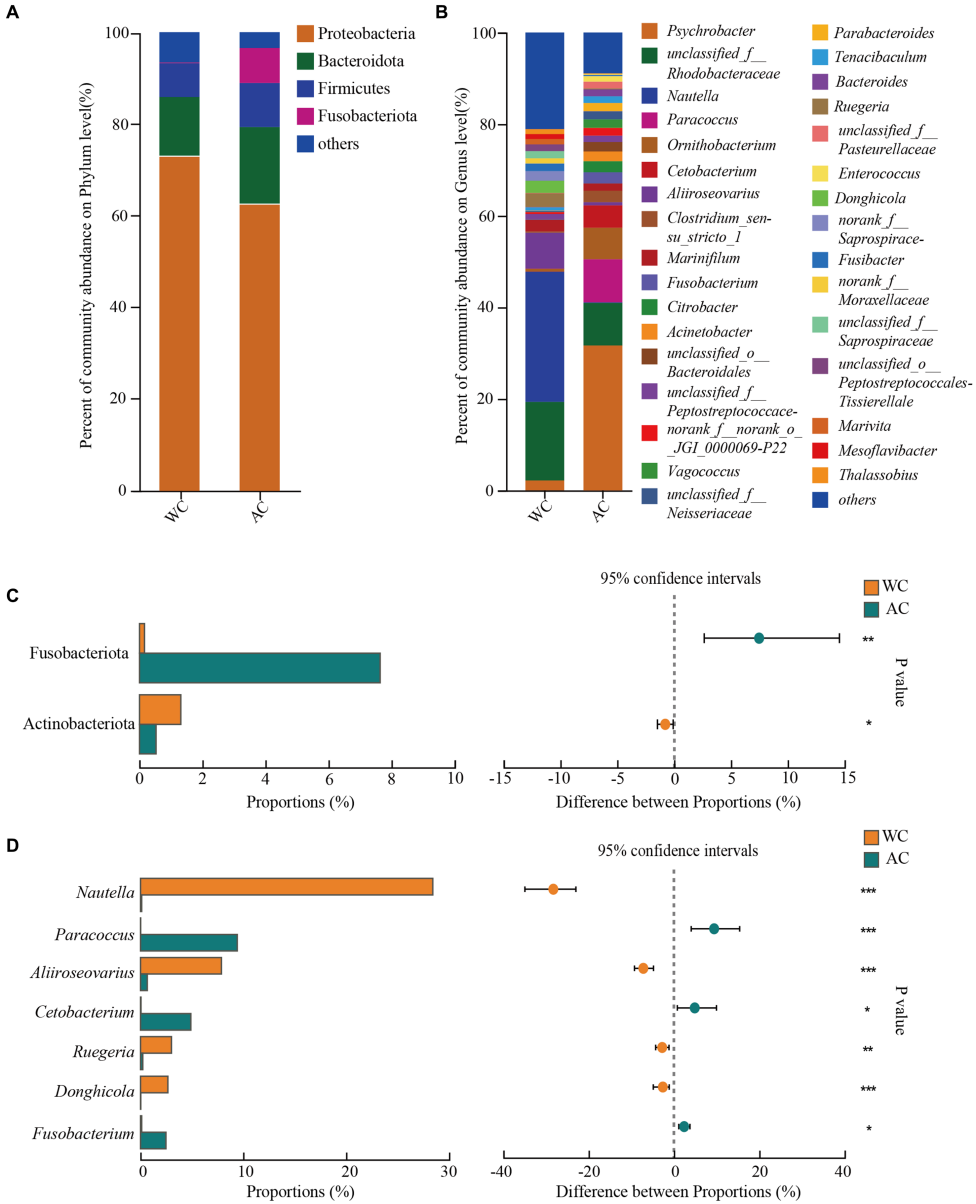
Comparative analysis of bacterial communities in wild and artificial breeding green sea turtles at the phylum and genus levels. **(A)** Display bar graphs representing bacterial compositions at the phylum level. **(B)** Display bar graphs representing bacterial compositions at the genus level. **(C)** Highlights differences in bacteria at the phylum level. **(D)** Highlights differences in bacteria at the genus level. Only phylum and genus with relative abundance greater than 1% are shown in this figure.

The dominant phyla (>1%) of intestinal bacteria in the wild and artificially bred green turtles comprised Proteobacteria, Bacteroidota, Firmicutes, and Fusobacteria (Figure 2A). Significant differences were observed in Fusobacteriota and Actinobacteriota between wild and artificially bred green turtles (Wilcoxon, *p* < 0.05; Figure 2C).

In wild green turtles, genera such as *Nautella*, unclassified Rhodobacteraceae, and *Aliroseovarius* comprised a higher abundance of intestinal bacteria. Conversely, in artificially bred green turtles, the predominant genera were *Psychrobacter*, *Paracoccus*, and unclassified Rhodobacteraceae (Figure 2B). The significant differences in the gut bacteria of wild and artificially bred green turtles were largely observed for *Nautella*, *Paracoccus*, *Aliiroseovarius*, *Cetobacterium*, *Ruegeria*, *Donghicola*, and *Fusobacterium* (Figure 2D).

**Figure 3:**
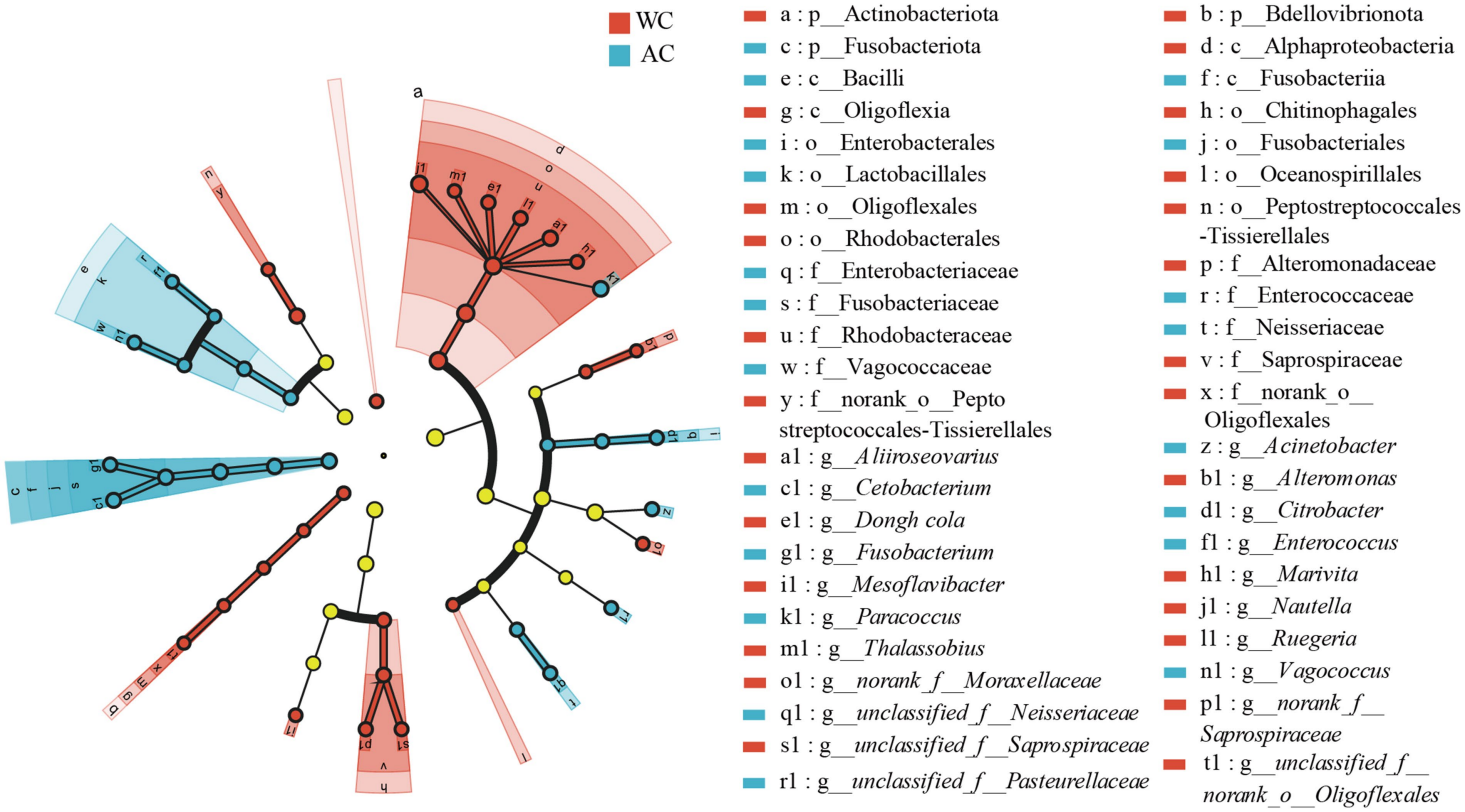
LEfSe analysis of wild versus artificial breeding gut bacterial biomarkers. Treatments are distinguished by colors for the WC and AC groups, with each circle diameter indicating abundance. Multiclass analysis allows for flexibility (at least one class difference). Inside-out circles represent taxonomical classification (from phylum to genus). Inside-out circles indicate the taxonomic classification from phylum to genus, with class, order, and family labels displayed. All taxa with LDA scores >3.5 are displayed.

Differences in the relative abundances of bacterial taxa between wild and artificially bred green turtles were determined based on LEfSe analyses. At the bacterial phylum level, the wild and artificially bred green turtles showed significant differences in Bdellovibrionota, Actinobacteria, and Fusobacteria (LDA > 3.5, *p* < 0.05; Figure 3). At the bacterial genus level, significant differences were observed in 21 genera between wild and artificially bred green turtles (LDA > 3.5, *p* < 0.05; Figure 3).

**Figure 4:**
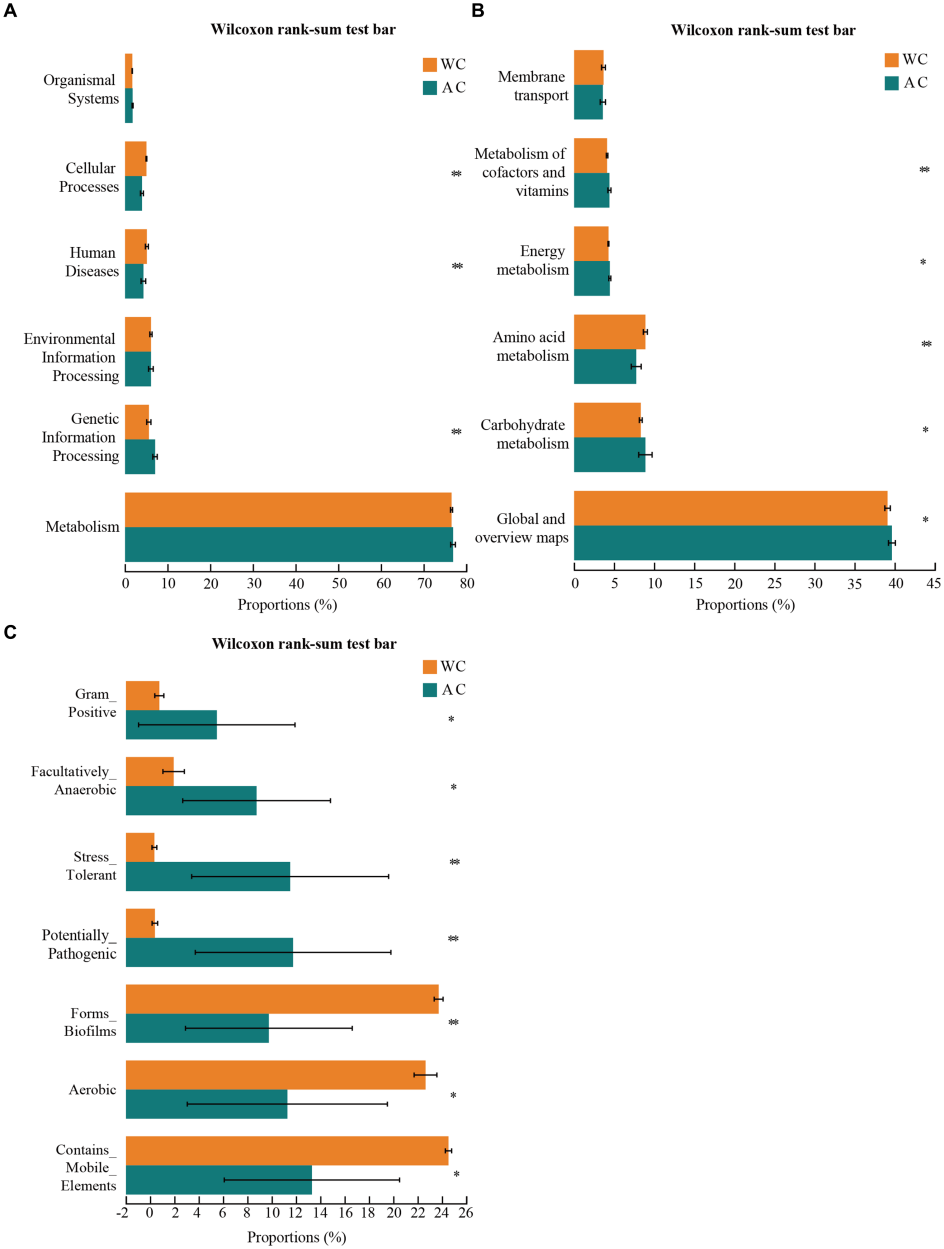
Functional analysis of bacteria was conducted using PICRUSt2. Bacterial phenotypes were identified using the BugBase method. **(A)** The relative abundance of predicted bacterial genes associated with level 1 KEGG pathways varies significantly across the macroscopic genome. **(B)** The relative abundance of predicted bacterial genes associated with level 2 KEGG pathways and functional pathways. **(C)** To determine functional and phenotypic differences between wild and artificially bred green turtle gut bacterial populations, bacterial phenotypes were analyzed and predicted using the BugBase algorithm, with significant intergroup differences denoted by *.

The bacterial microbiota in the guts of wild and artificially bred green turtles exhibited several key functions, including metabolism, genetic information processing, environmental information processing, human diseases, cellular processes, and organismal systems (Figure 4A). Wild and artificially bred green turtles exhibited significant differences in global and overview maps in carbohydrate, amino acid, energy, cofactor, and vitamin metabolism (Wilcoxon, *p* < 0.05; Figure 4B). To clarify the differential changes in gut bacterial flora between groups, we employed the BugBase algorithm to analyze and predict bacterial phenotypes. This allowed us to explore significant differences in the functions and characteristics of the gut flora, focusing on traits such as potentially_pathogenic and stress_tolerant (Figure 4C).

### 3.2 Comparison of the intestinal fungi of wild and artificial breeding green turtles

#### 3.2.1 rRNA sequencing analysis

The fungal assay produced 48,375–101,347 quality-filtered sequences per sample, amounting 1,681,307 sequences (477,514 reads in wild green turtles and 1,203,793 in artificially bred green turtles). The length distribution ranged from 230 to 255 bp (mean: 238 bp; Supplementary Table S3). All samples were sparse 47,868 reads for fungal sequencing (Supplementary Figure S3). Sparse curves reached the saturation stage, indicating that adequate sampling depth was achieved for each sample.

#### 3.2.2 α- and β-diversity analyses

**Figure 5:**
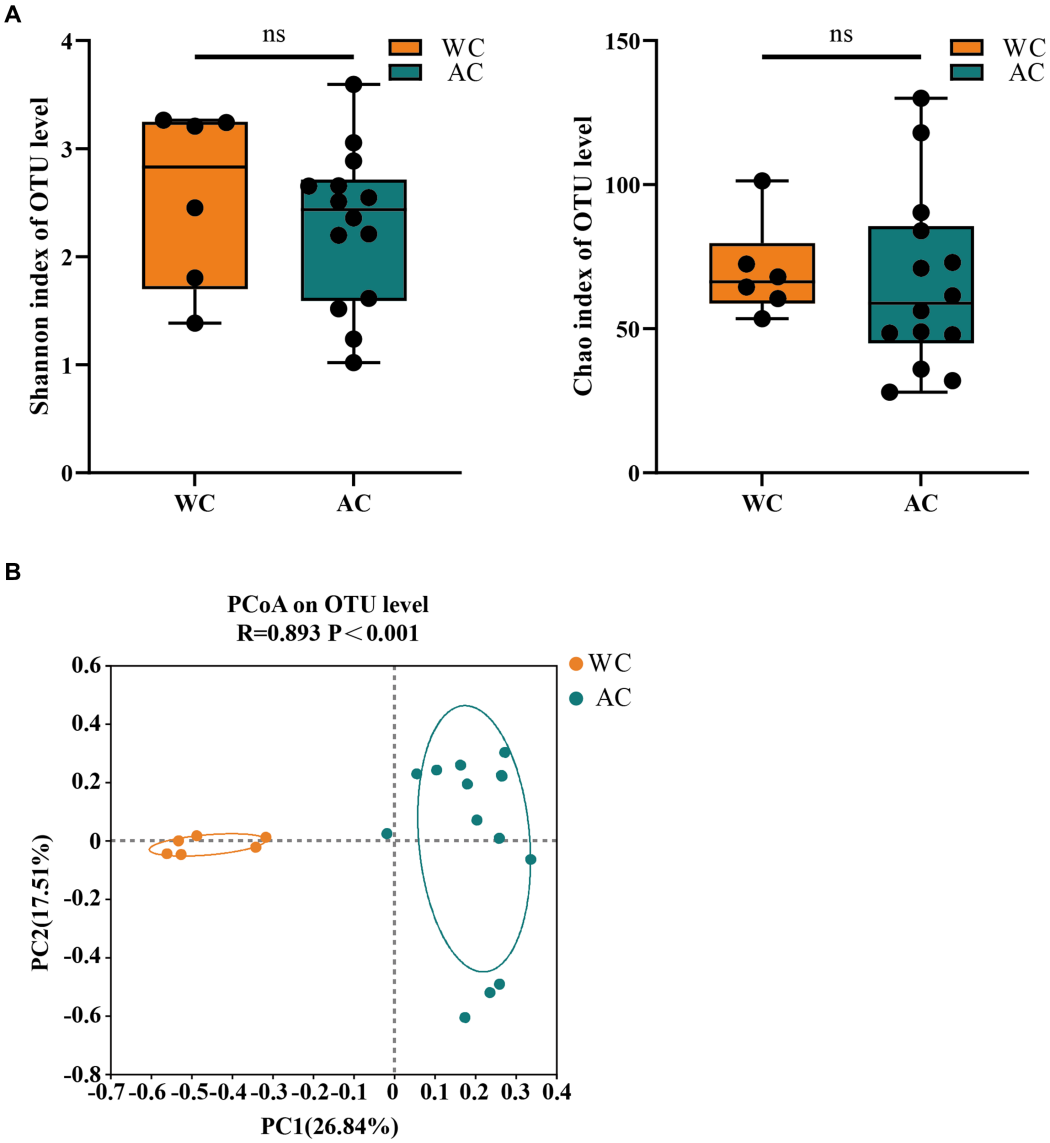
The α-diversity and β-diversity of gut fungi of the wild (WC) and artificial breeding (AC) green turtles. **(A)** Shannon and Chao data were not significant (*p* > 0.05) as determined via S-W and A-D tests, with normally distributed data. α-diversity of fungi was measured via Shannon and Chao indices. *p*-values indicate the confidence level of statistical analyses, with *p* < 0.05 indicating statistically significant differences. ** represents *p* < 0.01 and *** represents *p* < 0.001. **(B)** Principal component analysis (PCoA) plot of β-diversity based on fungi communities in anal swab samples of green turtles (*Chelonia mydas*). The principal coordinates (PC1 and PC2) are depicted in the axes, with percentages in parentheses indicating their relative contributions. Analysis was conducted using ANOSIM ANOVA. *R*-values range (−1 to 1), where *R*-values close to 0 indicate no significant differences between and within groups, while values close to 1 indicate greater between-group differences than within-group differences. *p*-values indicate the confidence level for statistical analyses, with *p* < 0.05 indicating a statistically significant difference.

Figure 5 shows the α-diversity indices (Shannon and Chao) for wild and artificially bred green turtles. β-diversity analysis was performed using principal coordinate analysis based on the Bray–Curtis distance algorithm (Figure 5). The ordination plot from cluster analysis revealed distinctly separated wild and artificially bred individuals.

The fungal community composition did not differ significantly between wild and artificially bred green turtles in Shannon and Chao indices (*p* > 0.05; Figure 5A). ANOSIM analysis revealed significant differences between the gut microbial communities of wild and artificially bred green turtles (*R* = 0.893, *p* < 0.001; Figure 5B).

#### 3.2.3 Microbial community composition and predicted functional analysis

Venn diagrams illustrate the shared and distinct fungal microbiota of wild and artificially bred green turtles (Supplementary Figure S4). Among the 600 OTUs in the fungal community, only 70 (11.67%) were shared. Among these, 50 OTUs belonged to Ascomycota (71.43% shared), 18 to Basidiomycota (25.71% shared), and 2 to unclassified_k Fungi (2.86% shared). Conversely, in wild green turtles, 152 OTUs were unique, with 62 belonging to unclassified_k__Fungi (40.79% unique), 56 to Ascomycota (36.84% unique), and 34 to the subphylum Basidiomycota (22.37% unique). For the artificially bred green turtles, 378 (63%) OTUs were unique, with 227 belonging to Ascomycota (60.05% unique), 77 to the subphylum Basidiomycota (20.37% unique), and 73 to the subphylum unclassified_k_Fungi (19.31% unique).

**Figure 6:**
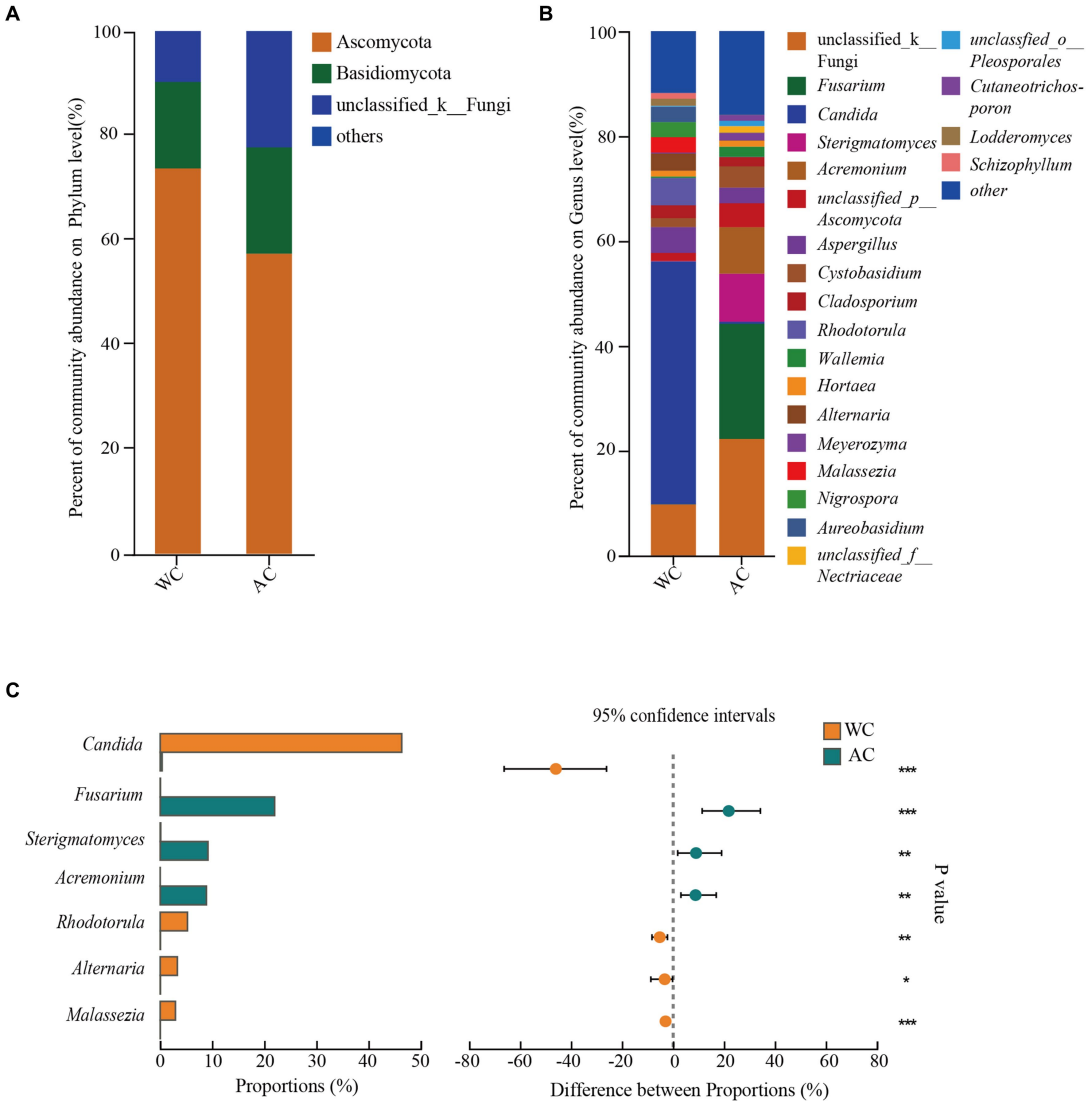
Comparative analysis of fungi communities in wild and artificial breeding green sea turtles at the phylum and genus levels. **(A)** Display bar graphs representing fungi compositions at the phylum level. **(B)** Display bar graphs representing fungi compositions at the genus level. **(C)** Highlights differences in fungi at the genus level. Only phylum and genus with relative abundance greater than 1% are shown in this figure.

The dominant intestinal fungal phyla (>1%) were Ascomycota, Basidiomycota, and unclassified_k_fungi (Figure 6A). No significant differences were observed between wild and artificially bred green turtles across these three phyla (Wilcoxon, *p* > 0.05). Meanwhile, in wild green turtles, the most abundant intestinal fungal genera were *Candida*, unclassified Fungi, and *Rhodotorula*; in artificially bred green turtles, unclassified Fungi, *Fusarium*, and *Sterigmatomyces* predominated (Figure 6B). Significant differences between wild and artificially bred green turtles gut fungi were observed in the genera *Candida*, *Fusarium*, *Sterigmatomyces*, *Acremonium*, *Rhodotorula*, *Alternaria*, and *Malassezia* (Figure 6C).

**Figure 7:**
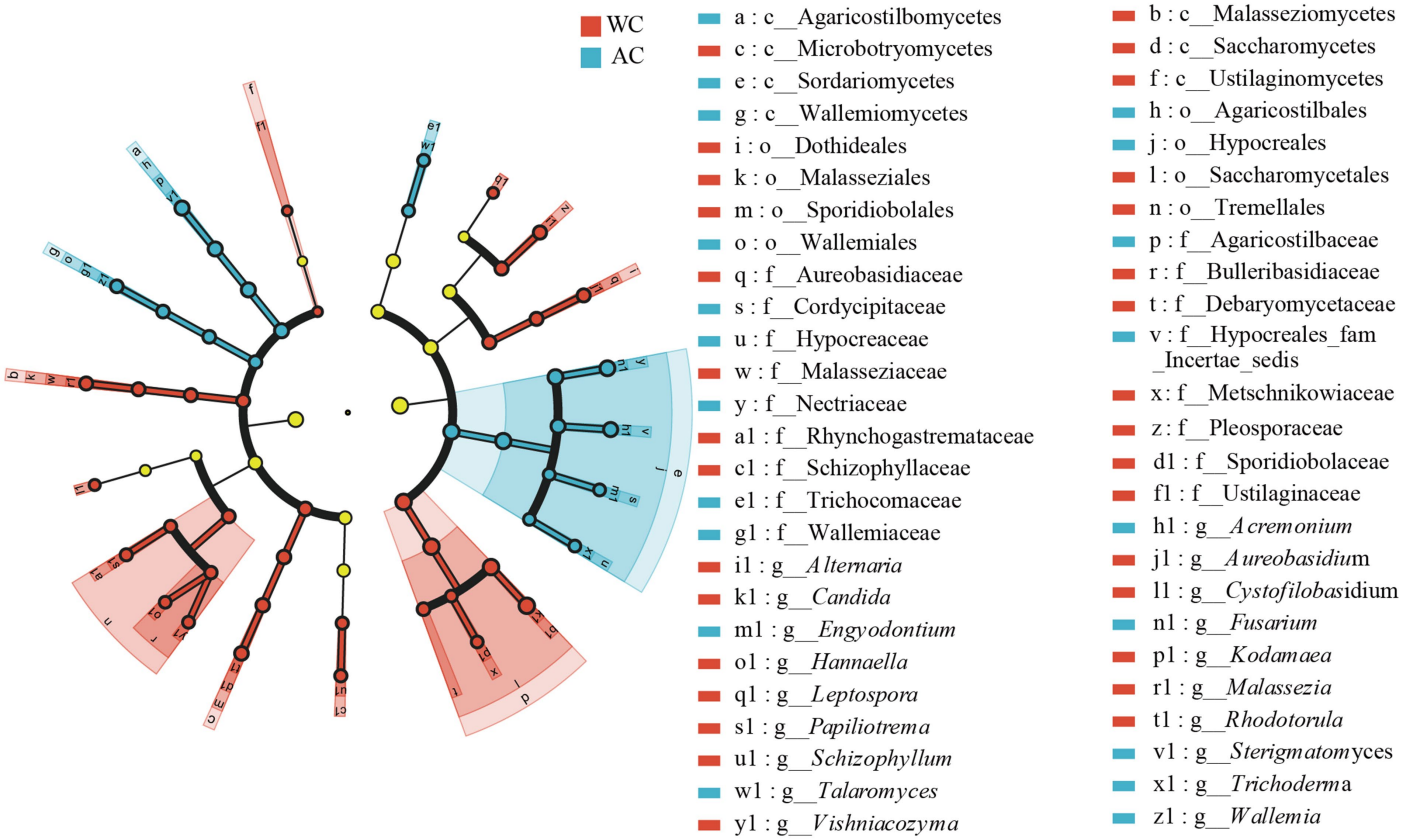
LEfSe analysis of wild versus artificial breeding gut fungi biomarkers. Treatments are distinguished by colors for the WC and AC groups, with each circle diameter indicating abundance. Multiclass analysis allows for flexibility (at least one class difference). Inside-out circles represent taxonomical classification (from phylum to genus). Inside-out circles indicate the taxonomic classification from phylum to genus, with class, order, and family labels displayed. All taxa with LDA scores >3.5 are displayed.

Differences in the relative abundances of bacterial fungal taxa between wild and artificially bred green turtles were determined based on LEfSe analyses. At the fungal phylum level, no significant difference was observed between wild and artificially bred green turtles (LDA > 3.5, *p* < 0.05; Figure 7). At the fungal genus level, significant differences were observed between wild and artificially bred green turtles in 19 genera (LDA > 3.5, *p* < 0.05; Figure 7).

**Figure 8:**
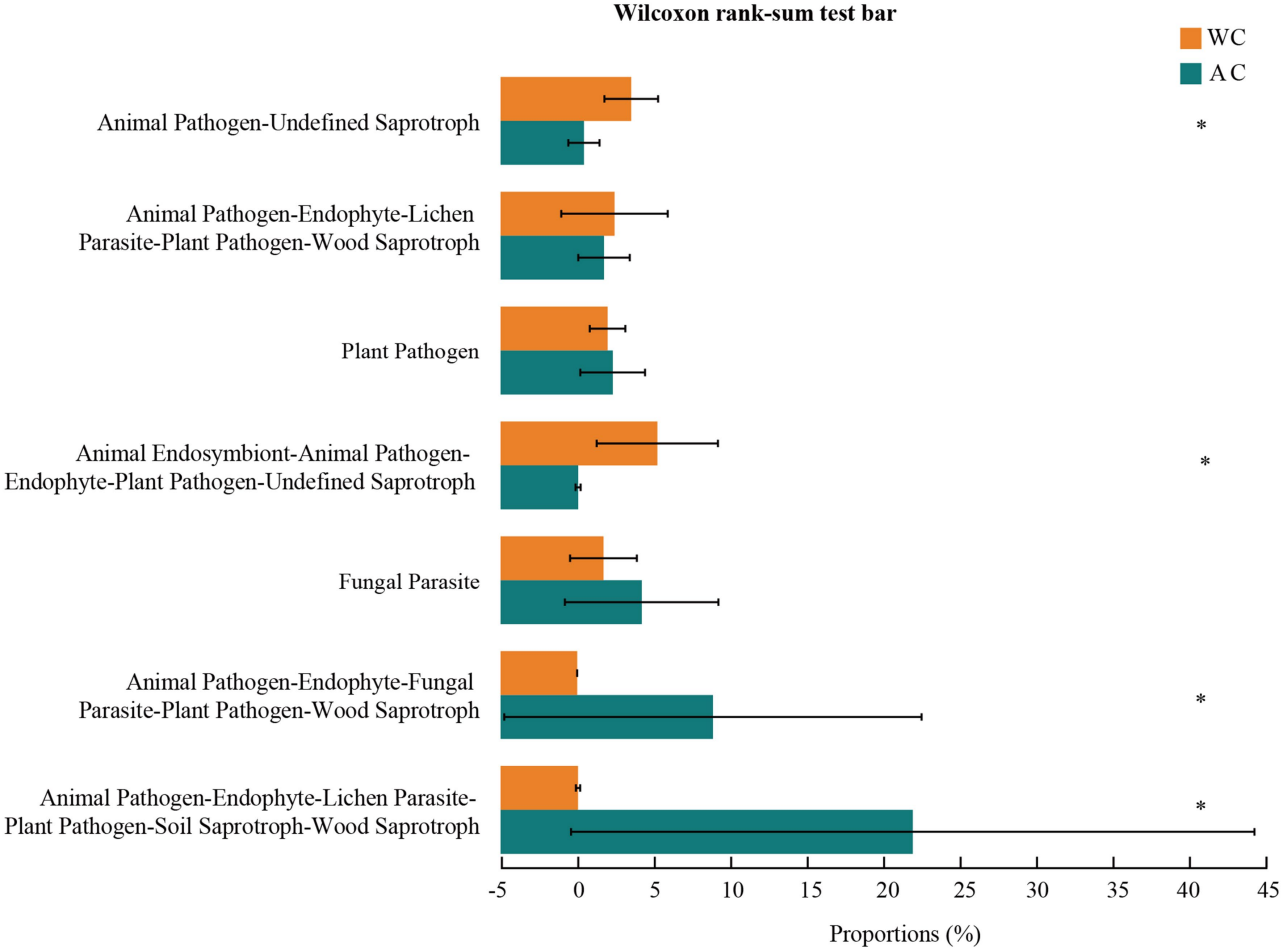
Functional analysis of fungi was conducted using FUNGuild. The potential functional taxa of the fungal community.

Fungal function prediction using Fungal Functional Guild showed significant differences in functions such as animal pathogens-endophytes-lichen parasites-plant pathogens-soil saprotrophs-sood saprotrophs between wild and artificially bred green turtles (Wilcoxon, *p* < 0.05; Figure 8).

## 4 Discussion

Understanding the relationship between beneficial and pathogenic gut microorganisms is crucial for animal health and disease management (Bäckhed et al., 2005). Exploring the gut microbiota of animals provides insight into their functions, changes, signaling pathways, and response mechanisms (Liu et al., 2023). Furthermore, modifying the gut microbiota can effectively prevent and treat certain diseases, thereby improving animal health, sustainable reproduction, and survival (Masry et al., 2021). Previous studies have demonstrated the importance of the gut microbiota in the health and disease of green turtles (Ahasan et al., 2018). In this study, we employed high-throughput sequencing to compare the gut microbial compositions of wild and artificially bred green turtles. Additionally, we assessed the health status of artificially bred green turtles by examining changes in their gut microbiota. These findings provide scientific guidance for releasing artificially bred green turtles and other wildlife species in nature.

Significant differences were observed between wild and artificially bred green turtles in bacterial community α- and β-diversity. Meanwhile, significant differences were only observed in the β-diversity of the fungal community, not in the α-diversity. The variations in bacterial communities were predominantly attributed to the diversity and relative abundance of Proteobacteria, Bacteroidota, and Firmicutes. Similarly, variations in fungal communities primarily stemmed from the diversity and relative abundance of Ascomycota, Basidiomycota, and unclassified Fungi. Wild green turtles exhibited a richer diversity of microbiota than artificially bred individuals, which is common in reptiles (Ma et al., 2008; Keenan et al., 2013) and fish (Ramírez and Romero, 2017; Tan et al., 2019). Ahasan et al. (2017) also showed that wild green turtles exhibit higher diversity in bacterial communities than stranded turtles, indicating possible dysbiosis in stranded turtles.

In this study, the relative abundances of Fusobacteriota and *Fusobacterium* were significantly higher in artificially bred individuals than in wild turtles. *Fusobacteria* spp. may be associated with inflammatory bowel diseases, such as ulcerative colitis and Crohn’s disease (Neut et al., 2002; Strauss et al., 2011). Specifically, *Fusobacterium nucleatum* and other *Fusobacterium* species induce a pro-inflammatory response in the host with virulence profiles that promote adhesion to and invasion of host epithelial cells (Bachrach et al., 2005; Uitto et al., 2005). Hence, artificially bred individuals may exhibit higher susceptibility to diseases, such as inflammation, than wild individuals.

Actinomycetes are widely found in the intestinal tracts of diverse animals (Bik et al., 2006; Ley et al., 2008), playing a vital role in host health. Actinomycetes species participate in balancing the internal environment of the intestinal barrier, enhancing nutrient absorption and promoting growth performance and immunity (Matsui et al., 2012; Binda et al., 2018). Thus, given that wild turtles exhibited a significantly higher abundance of Actinomycetes than artificially bred individuals, an immunological deficit might occur in the latter due to a less diverse or imbalanced gut microbiota. Consequently, the artificially bred turtles might be more susceptible to pathogens and diseases commonly found in natural habitats. Further research is needed to understand the specific mechanisms underlying this difference and develop strategies that could enhance the immune capabilities of artificially bred turtles, improving their chances of successful integration and long-term survival in the wild.

The marine genus *Nautella* exhibited higher abundance in wild individuals than in artificially bred turtles, owing to environmental differences between oceanic habitats and artificially bred habitats. Meanwhile, the denitrification potential of the genus *Paracoccus*, represented by the *Paracoccus* species, is frequently used to manage nitrogen pollution in aquaculture water. This could elucidate the substantial variation in the abundance of *Paracoccus* between wild and artificially bred individuals (Zhao, 2010). Additionally, *Paracoccus yeei* is classified as an opportunistic pathogen (Daneshvar et al., 2003) and is associated with various diseases, including peritonitis, bacteremia, corneal transplants, and heart transplants (Lasek et al., 2018). *Aliroseovarius* spp. are among the most abundant heterotrophic bacteria in marine environments, spanning seawater, sediments, algae, invertebrates, vertebrates, biofilms, and hypersaline microbial mats, as well as in aquaculture waters (Liu et al., 2022). *Donghicola*, a genus of bacteria typically found in seawater, primarily degrades tyrosine (Sung et al., 2015). Wild green turtles were significantly more susceptible to *Aliroseovarius* and *Donghicola* colonization than artificially bred green turtles, which may relate to the prolonged exposure of wild turtles to seawater habitats.

Artificially bred green turtles also had a higher abundance of *Fusobacterium* spp—a genus known for its association with various diseases and infections, including oral, head, and neck infections as well as localized skin ulcers (Shang and Liu, 2018). This poses a potentially significant ecological and public health concern. Releasing these artificially bred turtles into the wild could increase the abundance of Fusobacterium within the marine setting, introducing pathogens that may disrupt the delicate balance of the natural microbial community in wild turtles and potentially increasing their susceptibility to infection, affecting their overall health and impacting reproduction and survival rates. The consequences could be far-reaching, as a decline in the wild turtle population could disrupt aquatic ecosystems, affecting the biodiversity and natural balance of their habitats. Regarding human health, releasing artificially bred turtles with a higher pathogen load could pose risks. Contact between humans and these turtles or their environment allows zoonotic transmission of the pathogens carried by *Fusobacterium*. This could increase the incidence of human infections, particularly in those who are immunocompromised or engage in activities that cause them to be in regular contact with wildlife. Moreover, releasing such turtles could inadvertently introduce pathogens into the broader environment, affecting other species and possibly leading to unforeseen ecological impacts. This underscores the importance of pre-release health assessments and quarantine measures to minimize the risk of pathogen transmission to wild turtle populations and humans.

Commensal fungi constitute a small fraction of the gut microbiome but engage in diverse interactions with gut bacteria, including growth, nutrition, reproduction, and pathogenicity (Zhang et al., 2022; Maas et al., 2023). *Candida*—a commensal yeast in the oral cavity of healthy individuals—can also act as opportunistic pathogens. Non-albicans *Candida* species are gaining recognition as significant pathogens in human infections (Neppelenbroek et al., 2014). *Candida* levels were significantly higher in wild green turtles than in those artificially bred green turtles, possibly influenced by factors such as their living environment, diet, immune system, and genetics, warranting further investigation. Additionally, the abundance of *Fusarium* was significantly higher in the artificially bred green turtles than in wild green turtles. *Fusarium*—an important plant fungal pathogen (Ma et al., 2013)—can cause *Fusarium* diseases in humans and other animals (Alastruey-Izquierdo et al., 2008), such as corneal fungal infections (Gower et al., 2010). Hence, it may lead to *Fusarium* infections in green turtles and humans upon release into the wild. Wild green turtles also had significantly lower abundance of *Sterigmatomyces* spp. than artificially bred green turtles. *Sterigmatomyces*, a new fungal genus that includes *Sterigmatomyces halophilus*, can cause hepatic cysticercosis in humans (Imashioya et al., 2019). Hence, the difference in its abundance among green turtles may be influenced by the use of antibiotics to stimulate fungal growth in artificial breeding environments. To prevent diseases in artificially bred green turtles, antibiotics are administered to foster fungal growth. Indeed, the prevalence of *Acremonium* was also significantly higher in artificially bred green turtles than in wild green turtles. Several *Acremonium* species are opportunistic pathogens in humans and animals (Guarro et al., 1997). Human infections typically result from traumatic inoculation with the fungus, most commonly keratitis and pediculosis (Guarro et al., 1997). Thus, the *Acremonium* species in artificially bred turtles can pose a heightened risk of disease and human infections in wild green turtles. Meanwhile, *Rhodotorula*—similar to other types of yeasts—may competitively inhibit the growth of harmful bacteria by producing antimicrobial substances or occupying colony-forming positions, protecting the host from pathogens (Sako et al., 2023). Wild individuals exhibited significantly higher abundance of *Rhodotorula* than artificially bred individuals, potentially rendering wild individuals less susceptible to pathogen attacks.

The gut microbiome significantly influences the host through metabolic functions (Kuziel and Rakoff-Nahoum, 2022). We found that the main functions of the gut microbiota in wild and artificially bred green turtles were related to metabolism, genetic information processing, environmental information processing, human diseases, cellular processes, and organismal systems. These findings suggest the significant involvement of gut microbes in green turtle metabolism, which are similar to previous studies on turtle gut microbes (Ahasan et al., 2017). Additionally, correlations between green turtle gut microorganisms and human diseases were observed. The function and phenotype of gut flora in wild and artificially bred green turtles differ significantly regarding potential pathogenicity, further suggesting that artificially bred individuals may carry more potentially pathogenic bacteria. Fungal functions involve microorganisms contributing to the breakdown of organic materials in the digestive tract. However, most animal release activities are oversimplified, often entailing merely releasing animals into the wild. These actions overlook critical procedures such as health assessments and disease quarantine (Lin et al., 2023). For various reasons, released animals often die rapidly in the wild, leaving behind numerous carcasses that can contaminate the surroundings of the released sites. This directly threatens local ecosystems and human health (Lin et al., 2021). Simultaneously, releasing artificially bred animals without a quarantine period could pose a significant health risk to wildlife at the released site (Lu et al., 2020) and may lead to genetic contamination.

As we advance green turtle conservation, future research must evaluate the intricate symbiotic relationship between gut microbiota, the diet during the breeding process, and the environmental conditions. Genomic studies are fundamental to understanding the specific functions of gut microbes, which are essential for turtle health. Additionally, longitudinal studies will provide critical insights into how the gut microbiota affects the long-term survival of turtles once released into the wild, informing the development of pre-release strategies that promote ecological harmony. A primary focus will be on the influence of diet on the composition of gut microbiota and how this, in turn, affects disease resistance. The associated results will guide targeted nutritional interventions to bolster the turtles’ resilience. Simultaneously, the role of environmental factors in shaping the microbial balance and overall health of the turtles cannot be overlooked. To effectively address these complex issues, an interdisciplinary approach is needed. By integrating insights from microbiology, ecology, nutrition, and genetics, multifaceted conservation strategies can be devised that consider the intricate interplay between diet, environment, and gut microbiota. Employing a data-centric and innovative strategy that draws on the expertise of these diverse fields will be crucial in unlocking the full potential of the gut microbiome and developing more effective and ecologically responsible breeding and reintroduction programs. In this manner, we can support the continued survival and success of green turtle populations in their natural habitats.

## 5 Conclusion

This evaluation of the gut microbiota in wild versus artificially bred green turtles yields significant findings related to the health and conservation of species. The elevated α-diversity of intestinal bacteria in wild turtles and pronounced β-diversity differences indicate that gut microbiota diversity is fundamental to turtle health. Additionally, a compositional divergence exists within the gut microbiota with significant implications for disease transmission between artificially bred and wild green turtles. This emphasizes the necessity for rigorous health monitoring and the development of targeted interventions to improve disease resistance in artificially bred green turtles, ultimately protecting wild populations.

In conclusion, our research advances conservation strategies for green turtles by emphasizing the role of gut microbiota in health assessments and management while also providing a foundation for future research directions. Future studies should focus on elucidating the mechanisms underlying the observed differences in gut microbiota and exploring how these differences affect the turtles’ susceptibility to disease. Moreover, the long-term impact of gut microbiota on the health and ecological integration of reintroduced turtles must be established. By addressing these questions, we can refine our approach to artificial breeding and reintroduction programs, ensuring the successful restoration of green turtles in their natural habitats.

## Data availability statement

The data presented in this study are deposited in the NCBI Sequence Read Archive (SRA) under accession number PRJNA1091446, 1091539 (https://www.ncbi.nlm.nih.gov/sra/?term=PRJNA1091446, 1091539).

## Ethics statement

The animal study was approved by the Animal Research Ethics Committee of Hainan Provincial Education Center for Ecology and Environment, Hainan Normal University. The study was conducted in accordance with the local legislation and institutional requirements.

## Author contributions

XN: Data curation, Formal analysis, Investigation, Methodology, Writing – original draft, Visualization. LL: Formal analysis, Project administration, Supervision, Writing – review & editing. TZ: Data curation, Formal analysis, Investigation, Writing –review & editing. XA: Data curation, Investigation, Project administration, Writing – review & editing. YL: Data curation, Investigation, Writing – review & editing. YY: Data curation, Investigation, Writing – review & editing. MH: Formal analysis, Writing – review & editing. HS: Formal analysis, Writing – review & editing. LD: Conceptualization, Methodology, Project administration, Supervision, Writing – review & editing, Funding acquisition, Resources.
