# Supplementary figures and images for: Comparison of the intestinal flora of wild and artificial breeding green turtles (Chelonia mydas)
Source: Front Microbiol. 2024 May 30;15:1412015. doi: 10.3389/fmicb.2024.1412015 (PMC11170157; doi:10.3389/fmicb.2024.1412015)

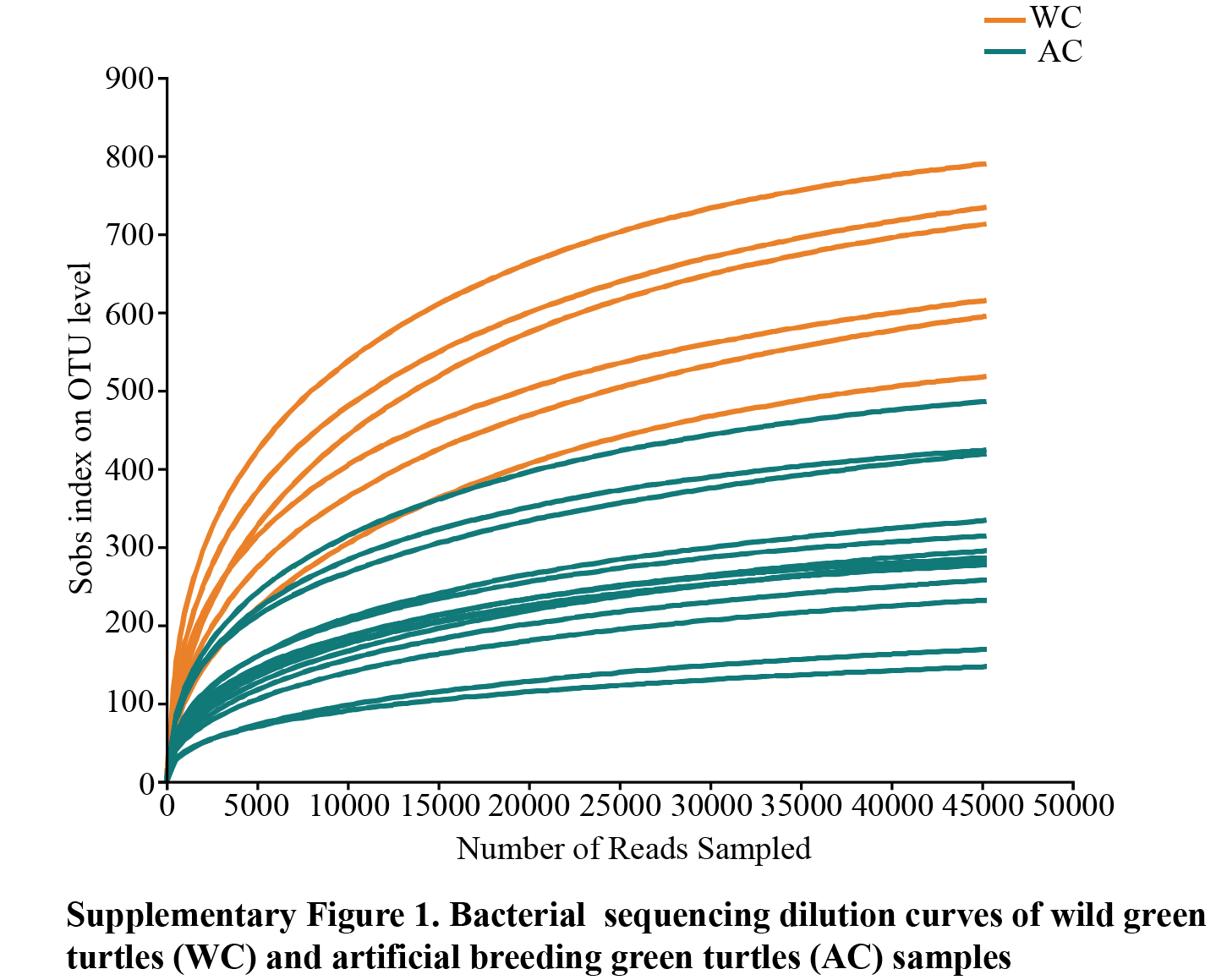

Supplement: Supplementary file 1 [file Image_1.TIF]

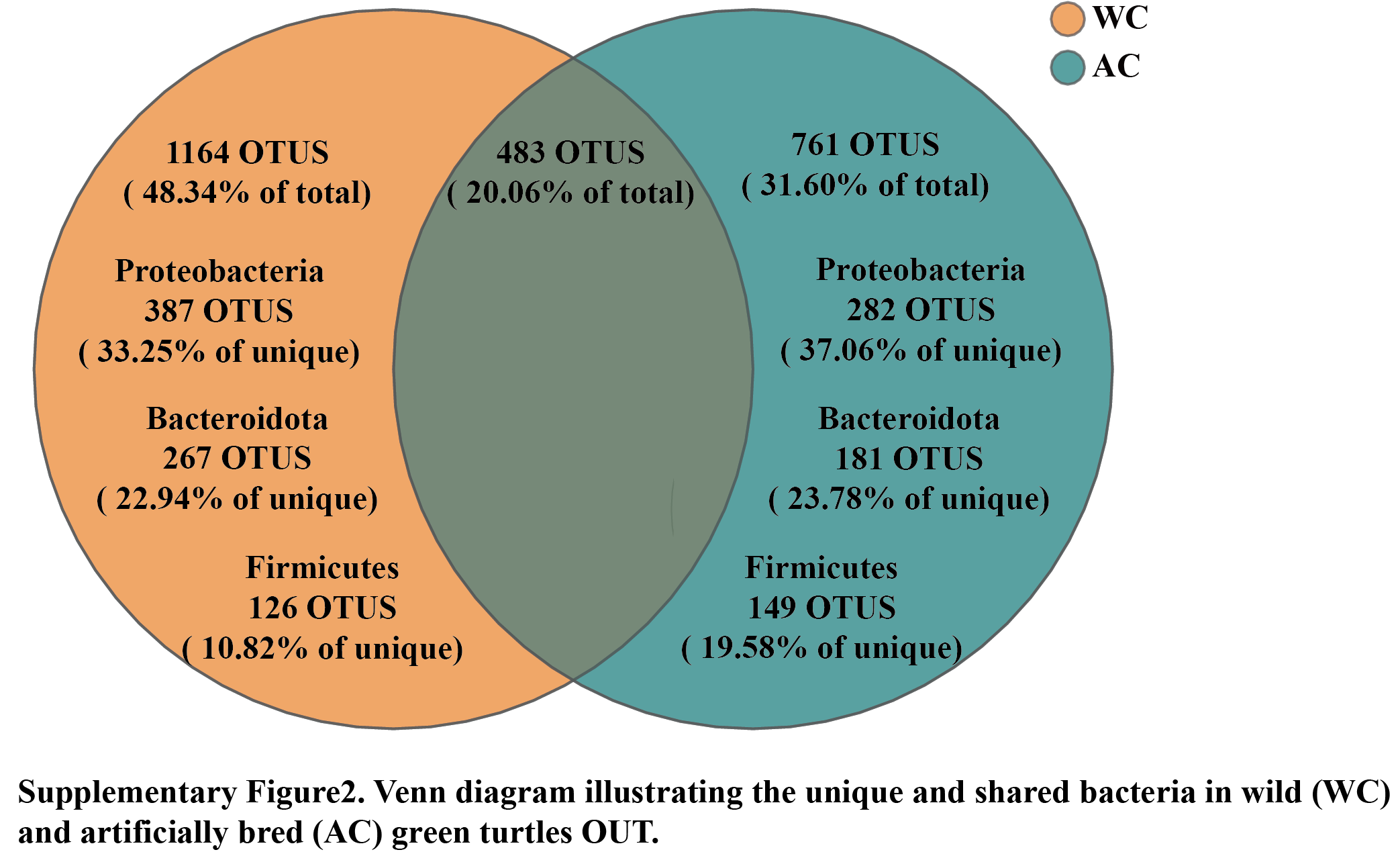

Supplement: Supplementary file 2 [file Image_2.TIF]

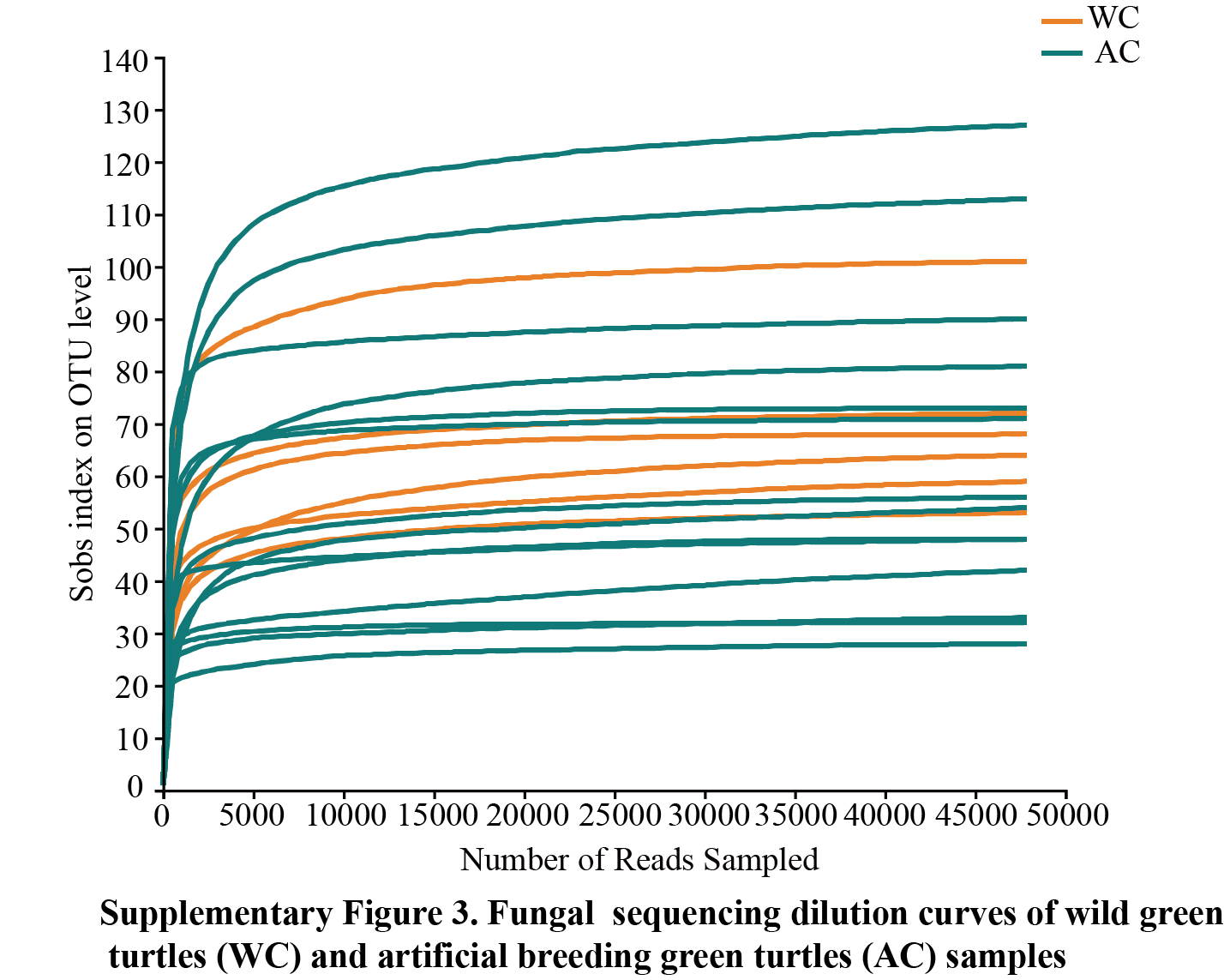

Supplement: Supplementary file 3 [file Image_3.TIF]

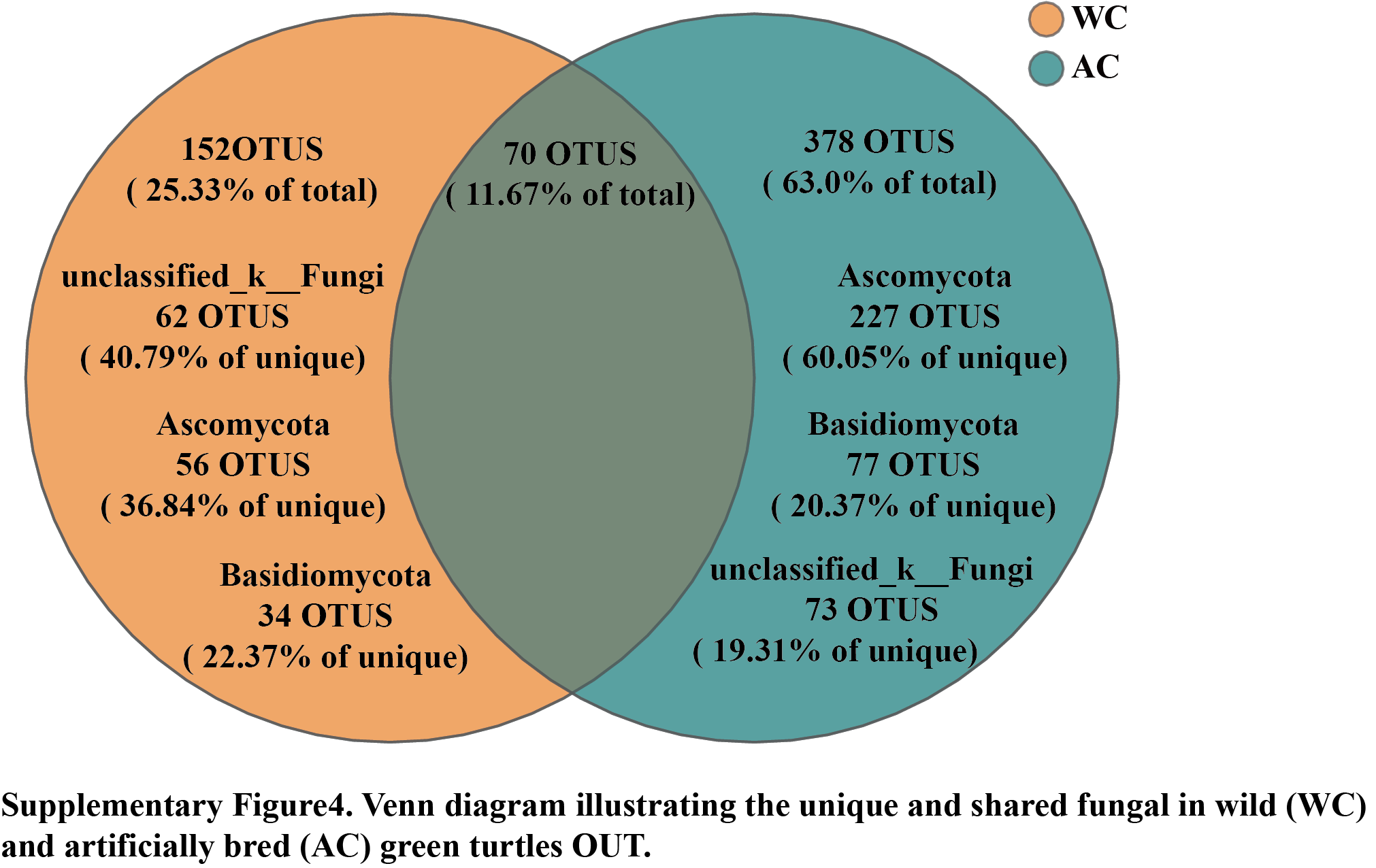

Supplement: Supplementary file 4 [file Image_4.TIF]
