# Supplementary material for: Comparison of the intestinal flora of wild and artificial breeding green turtles (Chelonia mydas)
Source: Front Microbiol. 2024 May 30;15:1412015. doi: 10.3389/fmicb.2024.1412015 (PMC11170157; doi:10.3389/fmicb.2024.1412015)
Supplement: Supplementary file 5 [file Table_1.DOCX]

**Supplementary Table 1**. Collection of basic information on individual sea turtles.

| Sample number | Weight/kg | Carapace length /cm | Carapace width /cm |
| --- | --- | --- | --- |
| WC01 | 72 | 53.1 | 19.8 |
| WC02 | 75 | 52.9 | 20.5 |
| WC03 | 80 | 54 | 22.5 |
| WC04 | 70 | 52.2 | 18.5 |
| WC05 | 76 | 56.9 | 25.5 |
| WC06 | 81.2 | 59.9 | 27.5 |
| AC01 | 22.5 | 20.5 | 1.79 |
| AC02 | 23.5 | 21.25 | 2.31 |
| AC03 | 24.5 | 21.5 | 2.47 |
| AC04 | 25 | 20.25 | 2.27 |
| AC05 | 25.3 | 20.5 | 2.39 |
| AC06 | 26.5 | 22.5 | 2.51 |
| AC07 | 27.5 | 23.5 | 2.9 |
| AC08 | 28.5 | 25.5 | 3.88 |
| AC09 | 29 | 26.5 | 3.85 |
| AC10 | 30.5 | 26 | 4.5 |
| AC11 | 31.5 | 27.5 | 4.46 |
| AC12 | 34 | 29.25 | 4.91 |
| AC13 | 36 | 30.5 | 5.89 |
| AC14 | 37.5 | 30.5 | 6.62 |
