# Supplementary material for: Comparison of the intestinal flora of wild and artificial breeding green turtles (Chelonia mydas)
Source: Front Microbiol. 2024 May 30;15:1412015. doi: 10.3389/fmicb.2024.1412015 (PMC11170157; doi:10.3389/fmicb.2024.1412015)
Supplement: Supplementary file 6 [file Table_2.DOCX]

**Supplementary Table 2**. Number and length of bacterial sequences in the samples.

| Sample\Info | Seq_num | Base_num | Mean_length | Min_length | Max_length |
| --- | --- | --- | --- | --- | --- |
| WC01 | 70002 | 28662297 | 409.45 | 232 | 433 |
| WC02 | 66766 | 27351717 | 409.67 | 277 | 444 |
| WC03 | 73409 | 30161952 | 410.88 | 317 | 490 |
| WC04 | 69698 | 28507110 | 409.01 | 317 | 431 |
| WC05 | 71425 | 29491744 | 412.91 | 206 | 432 |
| WC06 | 57055 | 23400871 | 410.12 | 262 | 492 |
| AC01 | 74239 | 30765463 | 414.41 | 261 | 528 |
| AC02 | 70489 | 29147873 | 413.51 | 302 | 438 |
| AC03 | 70610 | 29032845 | 411.17 | 233 | 430 |
| AC04 | 75193 | 31139625 | 414.13 | 202 | 439 |
| AC05 | 70374 | 29465305 | 418.70 | 224 | 433 |
| AC06 | 55753 | 23050884 | 413.45 | 239 | 431 |
| AC07 | 71078 | 29919350 | 420.94 | 206 | 433 |
| AC08 | 69347 | 29296033 | 422.46 | 294 | 433 |
| AC09 | 56763 | 24268287 | 427.54 | 217 | 437 |
| AC10 | 69750 | 28896837 | 414.29 | 317 | 431 |
| AC11 | 65062 | 27299155 | 419.59 | 204 | 433 |
| AC12 | 62797 | 26832543 | 427.29 | 223 | 435 |
| AC13 | 53255 | 22788320 | 427.91 | 392 | 435 |
| AC14 | 56025 | 23954632 | 427.57 | 213 | 431 |
