# Supplementary material for: Comparison of the intestinal flora of wild and artificial breeding green turtles (Chelonia mydas)
Source: Front Microbiol. 2024 May 30;15:1412015. doi: 10.3389/fmicb.2024.1412015 (PMC11170157; doi:10.3389/fmicb.2024.1412015)
Supplement: Supplementary file 7 [file Table_3.DOCX]

**Supplementary Table 3**. Number and length of fungal sequences in the samples.

| Sample\Info | Seq_num | Base_num | Mean_length | Min_length | Max_length |
| --- | --- | --- | --- | --- | --- |
| WC01 | 83334 | 19476207 | 233.71 | 151 | 359 |
| WC02 | 82661 | 19450913 | 235.31 | 145 | 411 |
| WC03 | 88415 | 20595822 | 232.94 | 138 | 437 |
| WC04 | 87572 | 21114107 | 241.11 | 142 | 423 |
| WC05 | 48375 | 12170310 | 251.58 | 120 | 425 |
| WC06 | 87157 | 20231931 | 232.13 | 60 | 381 |
| AC01 | 83964 | 21335219 | 254.10 | 136 | 403 |
| AC02 | 78023 | 19881007 | 254.81 | 136 | 403 |
| AC03 | 93043 | 23058320 | 247.82 | 135 | 403 |
| AC04 | 84126 | 19317463 | 229.63 | 50 | 411 |
| AC05 | 90845 | 21322896 | 234.72 | 60 | 369 |
| AC06 | 72833 | 17214042 | 236.35 | 85 | 358 |
| AC07 | 95102 | 22177537 | 233.20 | 57 | 384 |
| AC08 | 70496 | 17370956 | 246.41= | 170 | 411 |
| AC09 | 101347 | 23641342 | 233.27 | 149 | 370 |
| AC10 | 70941 | 16458986 | 232.01 | 104 | 375 |
| AC11 | 96499 | 22805929 | 236.33 | 136 | 418 |
| AC12 | 86774 | 20249816 | 233.36 | 77 | 394 |
| AC13 | 84619 | 19654833 | 232.27 | 143 | 337 |
| AC14 | 95181 | 22023647 | 231.39 | 142 | 377 |
